# Microdiversity characterizes prevalent phylogenetic clades in the glacier-fed stream microbiome

**DOI:** 10.1038/s41396-021-01106-6

**Published:** 2021-09-15

**Authors:** Stilianos Fodelianakis, Alex D. Washburne, Massimo Bourquin, Paraskevi Pramateftaki, Tyler J. Kohler, Michail Styllas, Matteo Tolosano, Vincent De Staercke, Martina Schön, Susheel Bhanu Busi, Jade Brandani, Paul Wilmes, Hannes Peter, Tom J. Battin

**Affiliations:** 1 Stream Biofilm & Ecosystem Research Lab, ENAC Division, Ecole Polytechnique Fédérale de Lausanne, EPFL, Lausanne, Switzerland; 2 Selva Analytics, LLC, Bozeman, MT USA; 3 Systems Ecology Research Group, Luxembourg Centre for Systems Biomedicine, University of Luxembourg, Esch-sur-Alzette, Luxembourg

**Keywords:** Climate-change ecology, Microbial ecology, Community ecology, Theoretical ecology

## Abstract

Glacier-fed streams (GFSs) are extreme and rapidly vanishing ecosystems, and yet they harbor diverse microbial communities. Although our understanding of the GFS microbiome has recently increased, we do not know which microbial clades are ecologically successful in these ecosystems, nor do we understand potentially underlying mechanisms. Ecologically successful clades should be more prevalent across GFSs compared to other clades, which should be reflected as clade-wise distinctly low phylogenetic turnover. However, methods to assess such patterns are currently missing. Here we developed and applied a novel analytical framework, “phyloscore analysis”, to identify clades with lower spatial phylogenetic turnover than other clades in the sediment microbiome across twenty GFSs in New Zealand. These clades constituted up to 44% and 64% of community α-diversity and abundance, respectively. Furthermore, both their α-diversity and abundance increased as sediment chlorophyll *a* decreased, corroborating their ecological success in GFS habitats largely devoid of primary production. These clades also contained elevated levels of putative microdiversity than others, which could potentially explain their high prevalence in GFSs. This hitherto unknown microdiversity may be threatened as glaciers shrink, urging towards further genomic and functional exploration of the GFS microbiome.

## Introduction

Glacier-fed streams (GFSs) are extreme ecosystems. In winter, they are characterized by darkness and ice, while in summer low temperatures but high UV-radiation and flow-induced hydraulic stress dominate the GFS environment, even with pronounced diel fluctuations [1]. While we increasingly understand that these ecosystems harbor diverse microbial communities [2–4], we do not know which phylogenetic clades in these communities are ecologically successful and what could underlie their success in a most extreme ecosystem.

**Fig. 1:**
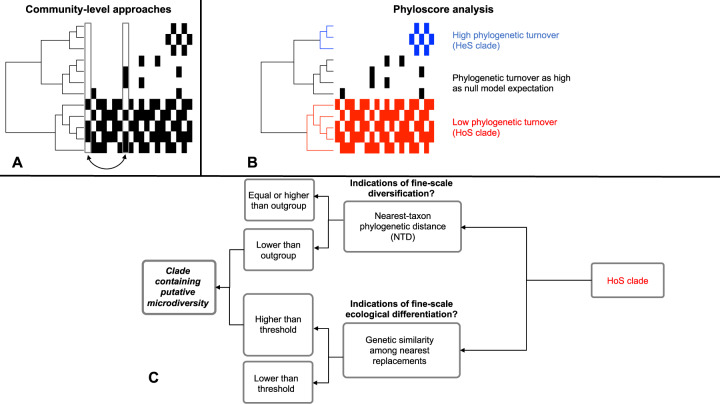
Community-wide and per-taxon phylogenetic turnover, and the subsequent search for putative microdiversity in specific phylogenetic clades. **A** A conceptual microbiome survey with the phylogenetic tree of the microbiome on the left and the respective presence/absence matrix on the right. Arrows indicate an example comparison such as it is currently performed with existing community-wide methods between two samples (columns), allowing comparisons across samples but not across clades. **B** The same survey, but analyzed following phyloscore analysis that allows comparisons of phylogenetic turnover across clades. The red clade (HoS clade) has high clade-wide prevalence; when a red taxon is not present other red taxa are present. This will result in low phylogenetic turnover (β-nearest taxon distances -βNTDs) clade-wise. Similarly, because the blue clade has low prevalence and the black clade is sparsely present in the ecosystem, the blue clade will have higher-than-expected phylogenetic turnover and the black clade will have similar phylogenetic turnover to the null model expectation. **C** HoS clades identified by phyloscore analysis can subsequently be examined for indications of microdiversity, by assessing the degree of within-clade fine-scale diversification and ecological differentiation.

Ecosystem-wide, ecologically successful clades should be more prevalent compared to other clades but there are currently no analytical tools to distinguish among clades with differential prevalence patterns. Because taxa within clades are phylogenetically related by definition, clade-wise phylogenetic turnover could be used to distinguish among such clades. In other words, an ecologically successful clade in GFSs should include taxa found across many GFSs and thus the phylogenetic distances of these taxa across different GFSs should be shorter compared to other clades. However, current methodologies calculate phylogenetic turnover at the community level, averaging across all taxa between any two given samples (Fig. 1A—between any two columns in the matrix) and making inferences about community assembly processes [5–7]. At the community level, higher-than-expected phylogenetic turnover between two communities indicates variability in environmental filtering (called “variable selection”), whereas lower-than-expected phylogenetic turnover indicates homogeneity in environmental filtering (called “homogeneous selection”) [5, 7]. While this approach can highlight the dominant ecological processes that underlie community assembly, it provides no indication on the contribution of individual taxa to the community-level turnover and so it cannot distinguish between clades with low or high phylogenetic turnover.

Here we developed a novel analytical framework that quantifies clade-wise phylogenetic turnover, “phyloscore analysis”, to detect clades with low phylogenetic turnover and therefore with high clade-wise prevalence in sediment biofilms of GFSs. Phyloscore analysis can identify clades with distinctly lower or higher phylogenetic turnover to that expected by chance and compared to other clades (Fig. 1B). For consistency with the existing frameworks [5, 7], we will henceforth call clades with distinctly low or high phylogenetic turnover as clades under homogeneous ecological selection (HoS clades) and clades under heterogeneous ecological selection (HeS clades), respectively (Fig. 1B).

We expected that, community-wide, low phylogenetic turnover (i.e., homogeneous selection) dominates in GFSs, likely as in other extreme and energy-limited ecosystems [8–10]. Therefore, we expected to find HoS clades driving this low phylogenetic turnover in GFSs, which we anticipate to be ecologically successful with broad spatial niches. Microbial clades with broad spatial niches are often hotspots of microdiversity [11–19], containing genetically similar sub-taxa, or *ecotypes* [20, 21], with distinct ecological niches [22]. Because of that, we additionally examined whether HoS clades in GFSs also show signs of microdiversity (Fig. 1C). To that end, we sampled biofilms from GFS sediments across a 340-km long transect in the Southern Alps in New Zealand (Supplementary Fig. 1), and analyzed the bacterial part of their microbiome using 16 S rRNA gene amplicon sequencing. Our sampling design allowed us to capture patterns of spatial community turnover over a large spatial scale as well as within GFSs where dispersal should be more important and could potentially attenuate selection by mass effects [7, 23] via the water flow.

## Materials and methods

### Sampling

We sampled 20 GFSs in the Southern Alps in New Zealand along a 340-km North-East – South-West transect (Supplementary Fig. 1). We selected GFSs from five major head valleys (Arthur’s Pass, Westland, Mount Cook, Mount Aspiring and Milford Sound). In each GFS, we sampled benthic sediments from two reaches (ca. 50 m long). The upper reaches (hereafter referred to as UP) were located the closest possible to the glaciers’ snouts, whereas the lower reaches (hereafter referred to as DN) were located 100–2500 m downstream from UP.

Within each reach, we sampled sediments from three patches to assess the within-reach variability; patches were 2–5 m apart. Wet sediment was sieved (315 and 250 µm, Retsch, Woven Wire Mesh Sieve) and the sandy fraction retained. All metal sampling material was flame-sterilized. Up to 30 g of sediment (in 10-ml cryovials, VWR) were flash-frozen in situ in liquid nitrogen pending DNA extraction. For bacterial abundance analysis, we filled 5-ml cryovials (VWR) with 2.5–3 grams of sediment containing a 10% solution of paraformaldehyde/glutaraldehyde [24] in 0.22 μm-filtered streamwater that we added in-situ, and we flash froze the vials in liquid nitrogen.

### Streamwater physicochemical parameters and sediment chlorophyll *α*

We measured streamwater temperature, dissolved oxygen and pH using a WTW Multi-parameter portable meter (MultiLine Multi 3630 IDS), electrical conductivity using a WTW—IDS probe (TetraCon 925) and turbidity using a WTW portable turbidity meter (Turb 430 IR) (Supplementary Table 1). Sediment chlorophyll *α* content was determined following a modified ethanol extraction protocol [25].

### Bacterial abundance

We quantified the number of cells per gram of dry sediment using flow cytometry after detaching the cells from the sediment matrix, by slightly modifying the method of Amalfitano & Fazi [26] as described elsewhere [25]. We identified and gated the cell populations based on the height of their fluorescence signals on a 530/30 – 725/40 nm biplot [27] (Supplementary Fig. 2), using the ACEA NovoExpress software with thresholds of 300 and 3000 on the front scatter and 530/30 nm channels, respectively. We analyzed three stained technical replicates plus one unstained replicate of the same extract per sample, the latter to exclude any background fluorescence. The coefficient of variation among the counts from technical replicates was 7.5 ± 5.1% on average. Finally, we corrected the acquired numbers for the various dilution factors and for the sediment water content, which we obtained from the weight loss of oven-dried sediment samples.

### DNA extraction, PCR amplification and 16 S rRNA gene amplicon sequencing

We extracted DNA from sediment samples using a phenol-chloroform method with certain modifications to address the nature of our samples [28]. We amplified the V3-V4 hypervariable regions of the bacterial 16 S rRNA gene using primers 341 f (5′-CCTACGGGNGGCWGCAG-3′) and 785r (5′-GACTACHVGGGTATCTAATCC-3′) that are general for amplifying Bacteria [29]. Due to low DNA yields and presence of inhibitors in the DNA extracts of certain samples and in an attempt to avoid PCR biases due to unequal input DNA, we diluted all DNA samples to a final concentration of ≤2–3 ng μl^−1^. The KAPA HiFi DNA Polymerase (Hot Start and Ready Mix formulation) was used in a 25-μl-amplification reaction containing 1X PCR buffer, 1 μM of each primer, 0.48 μg μl^−1^ BSA and 1.0 μl of template DNA. Amplification was performed in a Biometra Trio (Biometra) instrument. The thermal conditions applied after an initial denaturation at 95 °C for 3 min, were 94 °C for 30 s, 55 °C for 30 s and 72 °C for 30 s for 25 cycles followed by a final extension at 72 °C for 5 min. Amplification was verified on a 1.5% agarose gel and products were sent to Lausanne Genomic Technologies Facility (Switzerland) for further processing according to the MiSeq manufacturer’s protocol (https://support.illumina.com/documents/documentation/chemistry_documentation/16s/16s-metagenomic-library-prep-guide-15044223-b.pdf). In brief, a second PCR was performed for the addition of dual indices to the purified amplicon PCR products. This allowed extensive multiplexing of samples on a single sequencing lane of the MiSeq (Illumina) platform after quantification and normalization. Samples were sequenced using a 300 base paired-end protocol. Sequencing data has been uploaded to the European Nucleotide Archive under accession number PRJEB40567.

### Downstream sequence analyses

We used Trimmomatic v.0.36 [30] for quality filtering of the sequencing reads. Briefly, we truncated the reads in 4-base sliding windows at the first instance of mean quality dropping below a Phred score of 15, we removed the three leading and trailing nucleotides and we discarded the reads that were shorter than 200 bases.

We performed all subsequent sequence processing within the QIIME2 v.2019.1 framework [31]. We used DADA2 [32] with the default parameters to remove the primers, denoise and join the reads into exact amplicon sequence variants (ASVs). For this, 17 and 21 nucleotides (corresponding to the primers’ length) were removed at the beginning of the forward and reverse reads, respectively, and the reads were truncated at 300 bases. We performed denoising and joining of the reads using the default parameters, and we removed any ASVs that were not found in at least two samples. We used the alpha-rarefaction method implemented in the diversity plugin of QIIME2 to create the rarefaction curves (Supplementary Fig. 3). We used the ASV table that contained the raw sequence counts of each ASV at each sample to calculate the relative abundances of ASVs within samples, and we transformed the relative abundances into absolute abundances (cells per gram of dry sediment) by multiplying with the cell counts derived from flow cytometry [33].

We assigned taxonomy with the feature-classifier plugin [34] in QIIME2. First, we trained QIIME2’s naïve Bayesian classifier using the fit-classifier-naïve-bayes method on the Greengenes [35] 99% OTUs database v. 13.5. We created this training set using the extract-reads method with a minimal and maximal length of 250 and 550 nucleotides, respectively, and using the primers’ sequences. Finally, we assigned the taxonomy of the sequence variants using the classify-sklearn method with default parameters. We considered the taxonomies down to the genus level, ignoring “species” assignments that can be ambiguous based only on part of the 16 S rRNA gene [36]. Betaproteobacteria was the class with the highest relative abundance in all samples (Supplementary Fig. 4). A detailed taxonomic summary can be found in Supplementary Results (Supplementary Information—section “Detailed taxonomic diversity”).

To build the phylogenetic tree, we aligned the sequences of the ASVs with *mafft* [37] and we trimmed the alignment with the mask method in QIIME2 using the default parameters. We then used RAxML [38] with the GTRCAT substitution model and the rapid bootstrap option to build the tree, and the midpoint-root method to root the phylogenetic tree. To calculate pairwise nucleotide similarities we used ClustalOmega [39] v.1.2.3.

### Identification of the core microbiome

We identified the core microbiome across all samples based on taxonomy, i.e., as the consensus taxonomic clades that are present in all 40 reaches (20 GFSs x 2 reaches each). We used the package metacoder [40] in R [41] to visualize the results as hierarchy trees.

### Multivariate statistics

We used distance-based redundancy analysis to quantify the variance in the Bray-Curtis similarity matrix (calculated using the ASV table with log-transformed absolute abundances) that could be explained by the measured physicochemical variables, using the capscale function of the *vegan* [42] package in R. We performed a stepwise forward selection based on the increase in the adjusted *R*^2^ to select for the variables to include in the model, using the *ordiR2step* function in *vegan* with 200 permutations (Supplementary Table 2). The samples clustered in two major groups (Supplementary Fig. 5) while there was no single environmental variable driving this grouping (Supplementary Information—section “Environmental drivers of bacterial β-diversity”).

### Quantification of the dominant assembly processes at the community level

We used the framework developed by Stegen and colleagues [6, 7] to quantify phylogenetic and compositional turnover at the community level, which are indicative of the dominant community assembly processes. This framework assigns differences between two given communities (i.e., amplicons profiles of different patch samples in our case) to selection (either homogeneous or heterogeneous), to dispersal (either homogenizing or limiting) or to the lack of any dominant process. The influence of selection is first determined by examining the community-wise phylogenetic turnover between any two given communities via the *z* score (in this case called β-nearest taxon index - βNTI) of the observed β-mean nearest taxon distance (β-MNTD) from a null distribution of the same metric. The observed β-MNTD is calculated as the abundance-weighted mean of the nearest taxon distances for taxa that are present in only one of the two compared communities. The null distribution is created for the same pair of communities by shuffling the tips of the phylogeny, which essentially randomizes the presence/absence and abundances of taxa (in our case ASVs) in the compared communities but preserves the distribution of the phylogenetic distances. βNTI scores less than −2 indicate that the observed phylogenetic turnover is significantly lower than ~95% of the null values and thus that homogeneous selection between the compared communities causes higher-than-expected phylogenetic similarity (at short distances). In analogy, βNTI scores greater than +2 indicate the dominance of heterogeneous selection. Community pairs with βNTI scores between −2 and +2 are then further compared in terms of compositional turnover using the Raup-Crick distances based on the Bray-Curtis similarity (RC_Bray_), with the null distribution in this case being formed by probabilistic permutations under weak selection and random dispersal. This part of the analysis is based on the notion that passive dispersal should be blind as to the species’ phylogeny so it should result in non-distinguishable phylogenetic turnover from that expected by chance and in lower or higher compositional turnover (if it is homogenizing or limiting, respectively), from that expected by chance. Here, values of RC_Bray_ less than −0.95 and greater than 0.95 indicate less and more compositional turnover, respectively, than the null expectation and that is attributed to homogenizing dispersal in the former case and to dispersal limitation in the latter.

To apply the framework, two main assumptions must hold true for the examined dataset: (a) some degree of migration occurred among local communities at least at some point in evolutionary time and (b) phylogenetic conservatism exists, that is, phylogenetically more similar organisms occupy more similar ecological niches. For our dataset, the first assumption probably holds true even for the most distant GFSs because of migration via air, water flow and precipitation. To test the second assumption of phylogenetic conservatism we first calculated the niche optima of the ASVs for each physicochemical parameter that we included in the multivariate analyses (Supplementary Table 2), as previously described [43], and we then calculated the niche distances among ASVs as the euclidean distance of their niche optima (after standardization of each parameter). We subsequently performed a Mantel correlogram analysis, correlating the phylogenetic distances to the niche distances at different distance classes. Proper use of the βMNTD requires a positive correlation between the two at short genetic distance classes, indicating that at short phylogenetic distances more related ASVs have shorter niche optima distances and therefore occupy more similar ecological niches; that was indeed the case for our dataset (Supplementary Fig. 6). We calculated the abundance-weighted βMNTD using the comdistnt function of the picante [44] package in R (setting abundance.weighted = TRUE).

### Identification of phylogenetic clades under homogeneous ecological selection (HoS clades)

In addition to inferring community-wide patterns of assembly [6, 7], we developed a framework to identify HoS clades. In analogy to the community-wide framework, we defined HoS clades as monophyletic groups with distinctly low clade-wise phylogenetic turnover, i.e., groups containing ASVs with phylogenetically closer relatives across communities than expected by chance. Clades with such phylogenetic turnover patterns should have high niche occupancy across the examined samples (such as the red clade in Fig. 1B).

Our method consists of the following steps:

1. For a given pair of communities, *j*, *k*, and for each ASV, *i*, that is present in one but not both communities, we calculate its “phyloscore”. The phyloscore is a *z* score quantifying how different its nearest phylogenetic distance is to a null expectation in which species are randomly drawn to be present in the community in which ASV *i* is absent. For example, if we examine ASV *i* across communities *j* and *k* and *i* is present in community *j* and not in community *k*, we first find the nearest phylogenetic distance *d*_*i,j,k*_ of *i* based on the ASVs that are present in community *k*. We then sample a null distribution of *M* minimum phylogenetic distances $\left\{ {d_{i,j,k,m}^0} \right\}_{m = 1}^{m = M}$ between our focal ASV *i* and the ASVs present in community *k* in which ASV *i* is absent. If there are N_*k*_ species present in community *k*, we randomly sample N_*k*_ species other than ASV *i* to be present in the null community, compute the distance to the nearest present taxon to our focal ASV, and repeat this process *M* = 100 times to estimate the distribution of nearest phylogenetic distances in our null model. Finally, we calculate the phyloscore as:
  $$z_{i,j,k} = \frac{{\log \left( {d_{i,j,k}} \right) - \left\langle {\log d_{i,j,k,m}^0} \right\rangle _m}}{{\sigma _{i,j,k}^0}}$$
  where $\left\langle {\log d_{i,j,k,m}^0} \right\rangle _m$ is the average of the null distribution’s log-transformed nearest taxon distances and $\sigma _{i,j,k}^0$ the standard deviation of this distribution.
2. We then calculate for each ASV its total phyloscore as the sum of its phyloscores across all community pairs. We use phylofactorization [45, 46] to identify monophyletic clades of ASVs with significantly different total phyloscores compared to the complement set of ASVs and to extract the consensus taxonomic classification of the ASVs within. Phylofactorization is a graph-partitioning algorithm that sequentially cuts edges in a phylogeny, splitting the tree into disjoint sub-trees with high within-group similarities and between-group differences. At each iteration, phylofactorization cuts the edge that maximizes an objective function quantifying the difference between the sets of ASVs on either side of the edge. Here, the objective function was the absolute value of the t-statistic from a two-sample *t* test of equal variance on the total phyloscores between the two groups of ASVs on each side of the edge. The output of phylofactorization will be phylogenetic clades containing ASVs with distinctly different total phyloscores compared to outgroups. This second step is particularly important for distinguishing between niche-related patterns and dispersal. Dispersal would result in uniform phyloscores across all the phylogeny -high scores in the case of dispersal limitation and low scores in the case of homogenizing dispersal- whereas clade-specific high/low niche occupancy would result in monophyletic groups with lower/higher phyloscores, respectively, compared to outgroups.

We used the total phyloscore as an input for phylofactorization at the second step of the method to give more weight to ASVs that are frequently replaced by close relatives. However, this metric might be biased against ASVs that have only a few phyloscore values because of high or low occupancy even if all these values are negative. We therefore recommend the users to check the distribution of phyloscore values and decide on the appropriate metric. To facilitate this, the output of our online algorithm (https://github.com/sfodel/phylo_z_scores) includes all relevant per-ASV phyloscore values such as the sum (used here), the mean, the median, the number of values and the quotient of the mean and of the standard deviation. In our case, the choice of metric did not alter the results significantly (see “Results”).

Because the phylogenetic distance pool is preserved across all permutations, the phyloscores for each ASV are determined by presence–absence patterns alone and are independent of the branch lengths and patterns of speciation in the phylogeny. Because of that, we need to ensure that all potential sources of bias to the presence/absence matrix are excluded prior to the calculation of the phyloscores. To our perception the potential sources of bias can be either sequencing error biases or inadequate sampling biases. We describe below how we treated both these potential biases, and we recommend similar assessment in studies using our framework.

- Sequencing error biases. Sequencing errors can skew the distribution of presences/absences by inputting false positives, i.e., non-existent presences. Because these errors are more likely to happen in more abundant sequences, these false positives might tend to cluster around abundant ASVs in the dataset, artificially decreasing the phyloscores of abundant phylogenetic clades. To treat these potential biases we excluded ASVs observed in only one replicate sample. Taking into consideration the error correction implemented in DADA2 [32] with which we processed our sequencing data, we have no reason to assume that any residual errors are differentially distributed between HoS and non-HoS clades.
- Inadequate sampling biases. Inversely to sequencing errors, inadequate sampling biases can introduce false negatives (non-existent absences). In other words, low sequencing effort can be enough to capture all the diversity of abundant phylogenetic clades but not that of less abundant ones. In this way some ASVs in the latter clades can be left out and the presence/absence matrix can be artificially sparse in these clades. This concerns not only each HoS clade, but more importantly the outgroups against which these clades have distinctly different phyloscores. Thus, we needed to ensure that the non-HoS clades and each HoS clade are adequately sampled. For that we performed individual rarefactions for each of these phylogenetic groups and we observed that all such curves saturate, supporting that there was no bias due to inadequate sampling effort for any of the clades in question (Supplementary Fig. 7).

The R code for phyloscore analysis has been uploaded to GitHub (https://github.com/sfodel/phylo_z_scores).

### Assessment of putative microdiversity

We assessed putative microdiversity within each HoS clade and in non-HoS clades by searching for indications of fine-scale diversification and fine-scale ecological differentiation. This is not part of our developed phyloscore analysis, but rather complements it in the search for microdiversity within specific clades. Unlike other studies that focus on examining microdiversity within a-priori defined groups, e.g., within genera [47–49], here we assess putative microdiversity in HoS clades irrespectively of their breadth. We perceive this as an agnostic approach that can reveal even broad monophyletic groups with indications of fine-scale diversification and ecological differentiation.

To assess the degree of fine-scale diversification, we examined the clades’ structure by comparing the distribution of the nearest taxon distances (NTD—the shortest phylogenetic distance between a tip and all other tips) in each clade; fine-scale diversification within a clade should shift this distribution towards lower NTD values. Importantly, NTD examines shortest paths by definition and it should thus not be affected by how broad a given clade is, allowing comparisons among clades of unequal phylogenetic depth. This is an important aspect of NTD that can be leveraged to utilize the output of methods like phylofactorization that do not constrain the breadths of the identified clades. There was no relationship between a clade’s phylogenetic depth (distance from the root) and NTD for our dataset (Supplementary Fig. 8A). However, in simulations of randomly generated phylogenetic trees (1000 random trees of 1000 tips each, generated using the *rtree* function of the *ape* [50] package in R), we found a weak yet significant negative relationship between phylogenetic depth and NTD (Supplementary Fig. 8B). Users may therefore check for the existence of such a relationship in their datasets and correct the NTD values according to the clade’s depth if needed.

To search for indications of fine-scale ecological differentiation, we examined the nucleotide similarity among β-nearest ASVs in each clade HoS clade and in non-HoS clades. A β-nearest ASV of a focal ASV is the one that is most closely related phylogenetically to the focal ASV in a community where the focal ASV is not present. This analysis is complementing our phyloscore analysis in the search for microdiversity, because it further quantifies the genetic similarity among β-nearest ASVs that, by definition, is higher than expected by chance in HoS clades (because they have negative phyloscores) and significantly different compared to outgroups (i.e., to non-HoS clades, as identified by phylofactorization). We calculated this nucleotide similarity per ASV and we subsequently compared the distributions of the similarities within each HoS clade and in non-HoS clades. Similarity values of >97% indicate spatial replacements that occur among sub-taxa and are were thus used as an indication of the degree of fine-scale ecological differentiation of the respective clade.

## Results

### Homogeneous selection is the dominant assembly process at the community level

**Fig. 2:**
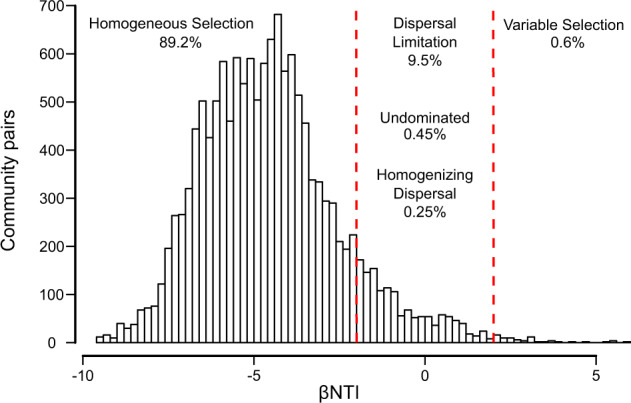
Homogeneous selection is the dominant assembly process at the community level. The histogram shows the distribution of βNTI values for sample comparisons across GFSs and the proportion of community pairs under each community assembly processes. Vertical dashed red lines are drawn at βNTI values of −2 and +2, which are the cutoff values for lower and higher than expected phylogenetic community turnover, respectively, the former indicating homogeneous selection and the latter indicating variable selection at the community level. The assembly processes governing the sample pairs in between are estimated from compositional turnover patterns based on the RC_Bray_ index.

Using a community-level framework [6, 7], we first examined the processes that govern community assembly among and within the GFSs. We found that homogeneous selection (reflected as βNTI values < −2) was the dominant assembly process for 89.2% of the community pairs among GFSs (Fig. 2). Moreover, homogeneous selection dominated (in 99.3% of the community pairs) the assembly within GFSs, indicating that it was not attenuated by downstream dispersal via water flow. Dispersal limitation drove assembly for 9.5% of community pairs among GFSs and its probability of occurrence increased with increasing geographic distance between the compared communities (logistic regression, *z* = 11.97, *p* < 0.001). Finally, variable selection and homogenizing dispersal drove assembly for 0.6% and 0.25% of community pairs among GFSs, respectively, while no single dominant process was found in 0.45% of community pairs.

### HoS clades are diverse, abundant and widespread in GFSs

Next, having confirmed the dominant role of homogeneous selection in driving assembly at the community level, we developed and applied a method that leverages null phylogenetic modeling to identify phylogenetic clades that are under homogeneous ecological selection (HoS clades—“Materials & methods”).

**Fig. 3:**
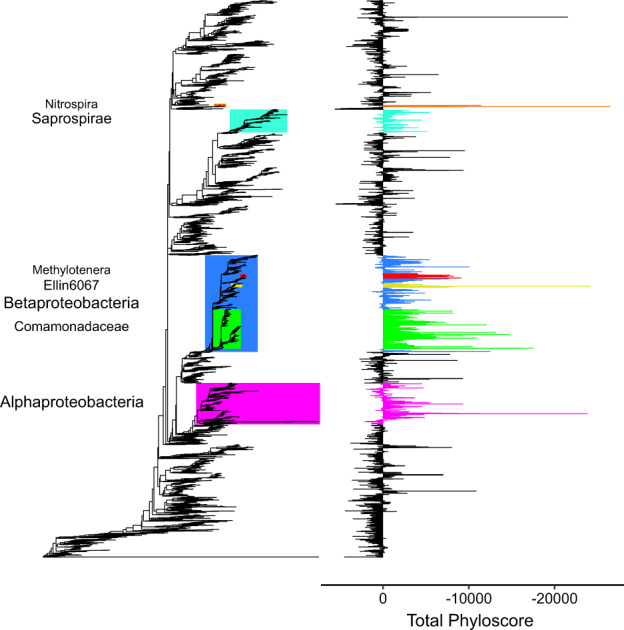
The identified phylogenetic clades under homogeneous ecological selection (HoS clades) that have lower within-clade total phyloscores compared to outgroups. HoS clades with >15 ASVs are color-coded on the phylogenetic tree, and the consensus taxonomy is given for each clade on the left with font size proportional to taxonomic depth. Clades nested within Betaproteobacteria are colored individually. The total phyloscore of each ASV (i.e., the sums of the phyloscores across community pairs) is shown to the right as bars with colors matching the clades’ colors.

We identified eight HoS clades with significantly lower total phyloscores compared to outgroups (contrast tests, 3.3E-16 < *p* < 6.8E-255), comprising of 5 to 1418 ASVs each (Fig. 3, Supplementary Table 3). The consensus taxonomy of the largest identified clade (1418 ASVs) affiliated to *Betaproteobacteria* (Fig. 3). This clade also contained three sub-clades with distinctly low total phyloscores and with consensus taxonomies affiliated to the family *Comamonadaceae* (575 ASVs), to the uncultured order Ellin6067 (54 ASVs) and to the genus *Methylotenera* (48 ASVs). The second largest clade (602 ASVs) had a consensus taxonomy affiliated to *Alphaproteobacteria* and it contained a low-score sub-clade (5 ASVs) affiliated to the genus *Novosphingobium*. The third largest clade (338 ASVs) was affiliated to the candidate class *Saprospirae* within *Bacteroidetes* while the smallest clade (18 ASVs) was taxonomically affiliated to the genus *Nitrospira*. Importantly, we did not identify any phylogenetic clade with significantly higher total phyloscores than expected by chance; this reflects the low contribution of heterogeneous ecological selection in governing assembly at the community level (i.e., low percentage of community pairs with higher-than-expected phylogenetic turnover; Fig. 2). The detected HoS clades were practically identical when we used the average, the median, or the quotient of the average and of the standard deviation of the phyloscores as alternative inputs for phylofactorization instead of the total phyloscores (Supplementary Table 4).

**Fig. 4:**
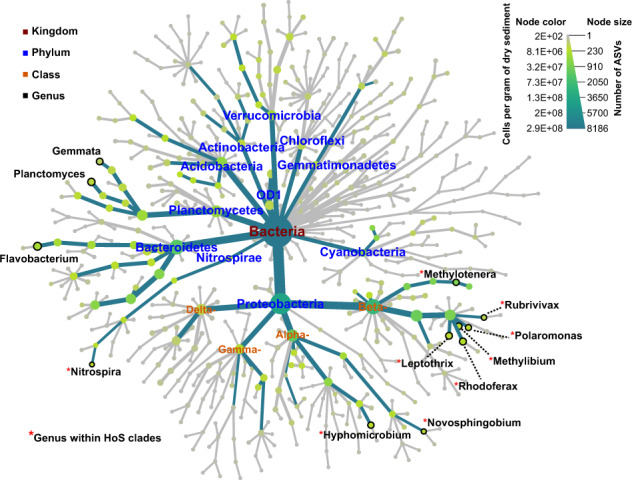
The core microbiome at the genus level and the HoS clades overlap highly. The overall core microbiome, i.e., taxonomic units found across all the sampled GFS reaches, is represented as a hierarchy tree (dark green edges) within the overall taxonomic tree (dark green and gray edges). The node color and size are proportional to the node’s abundance (cells per gram of dry sediment) and diversity (number of ASVs), respectively, as per the legend on the upper right. Only core genera, phyla, and classes within the Proteobacteria phylum are labeled to improve visualization with colors according to the legend on the upper left. Red asterisks indicate genera that reside in HoS clades.

**Fig. 5:**
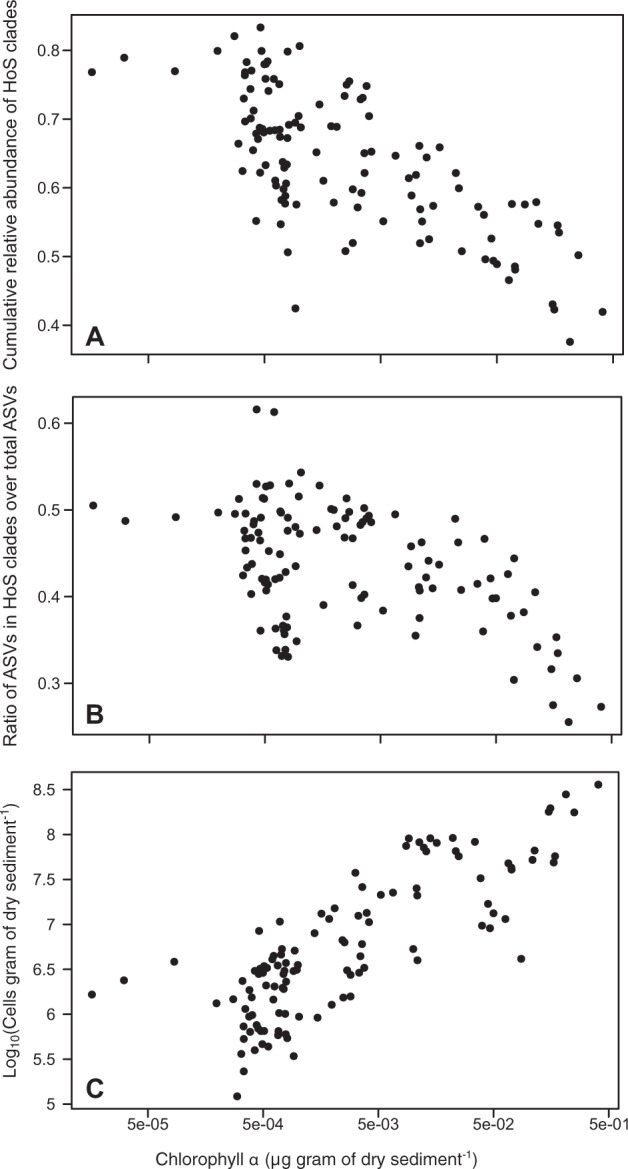
HoS clades thrive in sediments with low chlorophyll *a* that also have low total bacterial cell abundance. **A** The cumulative relative abundance of the HoS clades as a function of the sediment chlorophyll *a*. **B** The ratio of ASVs in HoS clades over the total ASVs as a function of the sediment chlorophyll *a*. **C** The total bacterial cell abundance as a function of the sediment chlorophyll *a*. For all panels, *n* = 119.

We found that HoS clades contained a significant part of the total bacterial α-diversity and abundance at all GFSs, with on average 43.7% (25.5–61.6%) of the total ASVs and 64% (37.6–83.3%) of the total sequences per sample. In addition, there was a notable overlap between HoS clades and the core bacterial genera, i.e., those genera present at all reaches (Supplementary Table 5, Supplementary Results). More specifically, nine of the twelve core genera resided within HoS clades (Fig. 4); these genera included the majority of the ASVs (59.5%) and of the sequences (87.7%) present in the core genera. Furthermore, both the abundance and the α-diversity of HoS clades increased disproportionately compared to the rest of the microbiome as sediment chlorophyll *a* decreased (linear models, *n* = 119, adjusted *R*^2^ = 0.3 and *p* < 0.001 for both models) (Fig. 5A, B). Since sediments with lower chlorophyll *a* also contained fewer total bacterial cells (Pearson correlation, r = 0.85, *p* < 0.001) (Fig. 5C), the above correlations held true with decreasing cell numbers as well (linear models, *n* = 119, adjusted *R*^2^ = 0.28 and 0.25, respectively, and *p* < 0.001 for both models) (Supplementary Fig. 9). Collectively, these results indicate that HoS clades are ecologically successful in the extreme GFS environment.

### HoS clades are hotspots for putative microdiversity

Finally, having identified the HoS clades, we examined whether they represent hotspots for putative microdiversity. For that, we searched for indications of higher fine-scale diversification and ecological differentiation in HoS clades compared to non-HoS clades. We examined the following two attributes in each HoS clade and in non-HoS clades: (a) the distribution of the NTD, expecting the presence of fine-scale diversification to shift this distribution towards lower values, and (b) the distribution of the nucleotide similarity among β-nearest ASVs, expecting the presence of fine-scale ecological differentiation to shift this distribution towards higher proportions with values >97% (i.e., indicative of spatial substitutions among sub-taxa).

**Fig. 6:**
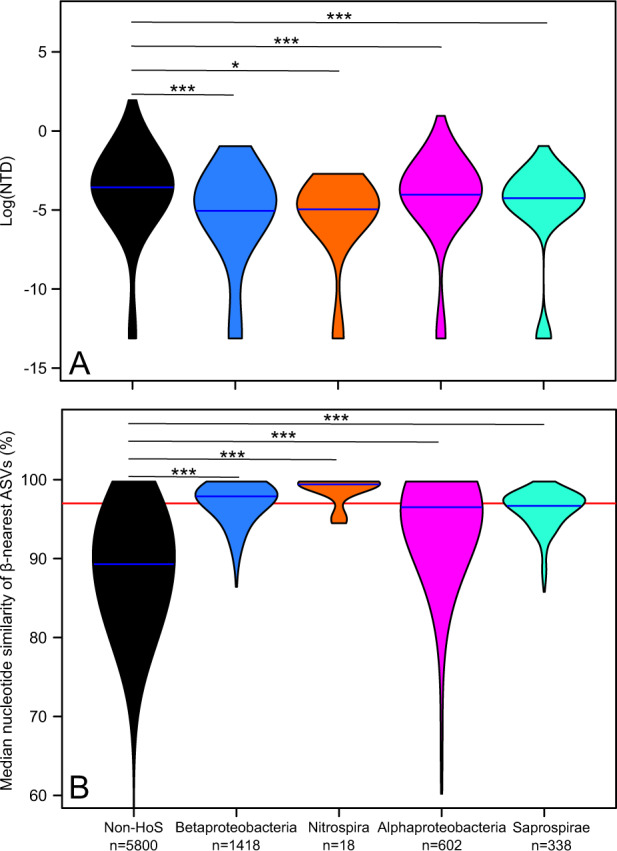
HoS clades are hotspots for microdiversity. **A** Violin plots showing the distribution of the nearest taxon distance (i.e., the phylogenetic distance between a given ASV and its closest relative—NTD, *y* axis in log-scale) in non-HoS clades and in each HoS clade. **B** Violin plots showing the distribution of the median nucleotide similarity of β-nearest ASVs (i.e., per-ASV median similarity between a given ASV *a* and its closest relatives in communities where *a* is not present, *y* axis in %) in non-HoS clades and in each HoS clade. Blue horizontal lines represent medians. Color-coding of HoS clades is as in Fig. 3. Asterisks correspond to the significance level of Wilcoxon tests (* and *** for levels of 0.05 and <0.001, respectively). The number of ASVs per clade (*n*) is shown at the legend on the bottom.

We found that the examined distributions were indeed shifted towards lower and higher values, respectively, in HoS clades compared to non-HoS clades (Wilcoxon tests, 0.03 < *p* ≪ 0.0001, Fig. 6). The lower NTD values in HoS clades compared to non-HoS clades (Fig. 6A) indicate that the ASVs within HoS clades have more similar closest relatives than the respective ASVs in non-HoS clades. In addition, the higher nucleotide similarity of the β-nearest ASVs in HoS clades compared to non-HoS clades (Fig. 6B) indicates that ASVs are spatially replaced by more similar ASVs within the former. Particularly important for the presence of microdiversity in HoS clades is the fact that 45.2–83.3% of these replacements occur among ASVs that are >97% similar, whereas this percentage is only 11.7% for non-HoS clades. Collectively, these results suggest that HoS clades represent hotspots of putative microdiversity.

## Discussion

### Ecologically successful and microdiverse clades in the GFS sediment microbiome

Using a novel analytical framework for phylogenetic turnover analysis, we detected prevalent and putatively microdiverse phylogenetic clades in the sediment microbiome of GFSs. Low phylogenetic turnover at the community level, attributed to homogeneous selection in community ecology [6, 7], dominated the assembly in the sampled GFSs as is typical for extreme environments [8–10]. Our analytical framework further allowed us to dissect the contribution of individual phylogenetic clades (HoS clades) to this low phylogenetic turnover. The high occupancies and abundances of HoS clades in GFSs corroborate that they are ecologically successful therein. Focusing on the phylogenetic structure and on the similarity among β-nearest taxa in HoS clades, we found signs for both higher fine-scale diversification and ecological differentiation compared to non-HoS clades. These findings shed new light on the presence of fine-scale phylogenetic architecture and its consequences for the success of microbial life in the extreme GFS environment.

Our results further suggest that unlike non-HoS clades, the identified HoS clades can successfully occupy a niche in GFSs that are largely devoid of algal primary producers. This is indicated by their stronger presence in sediments with low chlorophyll *a* content. Concomitantly, lower cell abundance in these sediments further evokes that the rest of the microbiome is energy-limited in these sediments. We interpret these patterns as evidence for an ecological niche governed by chemolithotrophic rather than heterotrophic energy pathways, as is typical in extreme environments like the cryosphere and the deep biosphere [51–54]. We note here, however, that cell detachment biases prior to flow cytometry are still undocumented for the particular ecosystem and might have affected the observed patterns.

This notion of metabolic versatility in HoS clades is indeed supported by the known physiologies of some of their genera. For instance, the globally-spread [55] psychrophilic genus *Polaromonas* is facultatively chemolithotrophic and metabolically versatile [56], and was even reported to be microdiverse [47]. Furthermore, the obligate methylotrophs *Methylibium, Methylotenera* and *Hyphomicrobium* have been found in deglaciated alpine soils [57] and glaciers [58], and can utilize a diverse array of C_1_ compounds [59–61] that can occur as intermediates in methane oxidation that is typical for the sub-glacial environment [62, 63]. Interestingly, the family *Methylophilaceae* that contains *Methylotenera* and *Methylibium* is one of the groups that has been reported to diversify quickly [64], supporting the existence of microdiversity therein as suggested by our analyses. The anoxygenic phototrophs and nitrogen fixing genera *Rhodobacter, Rubrivivax* and *Rhodoferax* include psychrotolerant isolates [65, 66] and have been found in ice cores [67], deglaciated soils [68] and glaciers [58, 69]. Furthermore, members of the *Nitrospira* genus are ubiquitous nitrite oxidizers, and species able to perform complete ammonium oxidation have recently been reported in a high-altitudinal and cold-water river [70]. The sulfur-oxidizing, facultative anaerobe and chemolithotrophic *Thiobacillus* has a sequenced genome from a subglacial isolate revealing cold adaptations [71] and is frequently found in cold-related environments [72, 73]. The only “classical” heterotroph among the identified genera is the iron oxidizing *Leptothrix* [74], which has been recently reported from a metagenome from Antarctica [75].

The environment of GFSs is predicted to change dramatically as glaciers shrink owing to climate change [1, 76]. A recent synthesis has suggested that specialist species that are well adapted to the glacial conditions in GFSs are highly threatened by glacier retreat [76]. At the same time, as turbidity decreases in GFSs because of reduced discharge and sediment loads, the environment will become more advantageous for primary production [1]. Therefore, the ecological niche with its putatively microdiverse clades that we have identified will most likely vanish with ongoing glacier shrinkage, and with this, a hidden biodiversity that has adapted to the GFS environment and that could even contain unexploited potential for biotechnology [77].

Contrary to the early expectations of an ecologically neutral origin of microdiversity arising from genetic drift [78], the stronger presence of putative microdiversity in HoS clades than in non-HoS clades suggests that optimization of niche occupancy could underlie the observed microdiversification in GFSs. We conjecture that this could be a phenomenon common to the microbiome of other extreme environments that might have been hitherto unrecognized because of the lack of adequate analytical frameworks and of the knowledge of relevant ecological gradients. The relaxation of the current selective constraints owing to climate change may change the balance among the selective processes in GFSs and that could erode the microdiversity of the GFS microbiome with yet unknown consequences for the overall biodiversity and ecosystem functioning therein.

### Phyloscore analysis

Our developed framework, phyloscore analysis, can be used to explore microbiomes for putative hotspots of microdiversity even in the absence of known ecological gradients or isolates as often required previously [16, 18, 19, 79]. Commonly, microdiversity is assessed by revealing distinct temporal niches among sub-taxa [80, 81], or by using differential abundance along environmental gradients [82] or differential co-occurrence patterns [49]. However, often it is hard to know or even quantify the ecological gradients of interest along which to look for microdiverse clades. Because of that, we might still be missing important microdiverse clades. In contrast, phyloscore analysis does not require such a priori knowledge. For example, *Synechococcus* in hot spring mats has been shown to develop microdiversity patterns along temperature, light and oxygen gradients [83, 84]. Even if we did not know the exact environmental gradients therein but we performed a microbiome survey and deployed phyloscore analysis, we could start looking for microdiversity in *Synechococcus* because we would detect it as an HoS clade.

We acknowledge that, because of the lack of phenotypic and genomic comparisons among taxa in the HoS clades, we cannot assess their degree of ecological differentiation. For example, in the presence of low environmental heterogeneity across a given dataset, HoS clades with low NTDs and high β-nearest genetic similarity could still comprise prevalent “generalist” taxa with fine-scale, yet ecologically neutral, diversification. In other words, microdiversity in a clade should result in “HoS-like” patterns but not all HoS clades are necessarily microdiverse even if they show low NTDs and high β-nearest genetic similarity. Such uncertainties are common to any similar method that lacks phenotypic characterization, like the Ecotype Simulation [85] that assigns putative ecotypes based on evolutionary simulations of nucleotide sequences. Thus, phyloscore analysis serves as a good starting point to screen for putatively microdiverse clades that can then be further examined for genomic and phenotypic differences.

Apart from being coupled to the search for microdiversity, phyloscore analysis can be used as a standalone tool to identify phylogenetic clades driving community assembly patterns, which is a debated topic in microbial ecology [86, 87]. Analytical frameworks detecting and quantifying assembly processes at the community level [6, 7, 88, 89] have provided useful insights in a great variety of ecosystems [87]. These frameworks identify dominant assembly processes, but in most cases multiple processes act simultaneously [90]. Thus, recently the focus has expanded to the identification of specific components of the microbiome that underlie community-level assembly processes. For instance, the recent iCAMP [91] forms phylogenetic bins of taxa, examines their phylogenetic and taxonomic turnover, and assigns the underlying processes governing their turnover. Our analytical framework is conceptually similar to iCAMP and can be used in parallel with it. Like iCAMP, our framework detects clades with distinctly different phylogenetic turnover than that expected by chance. The detected phylogenetic clades do not necessarily need to have low phylogenetic turnover like in the present study; clades with high phylogenetic turnover indicative of heterogeneous selection (i.e., disproportionally present in different sample groups like the blue clade in Fig. 1B) can be detected as well. Such patterns would indicate clades with niches in specific spatial or temporal subsets depending on the study. Unlike iCAMP, however, our method avoids phylogenetic binning and uses nearest-taxon phylogenetic distances. Both of these methodological attributes can be valuable when examining patterns near the tips of the identified phylogenetic clades, which might not emerge with the use of other metrics [6]. Nevertheless, the short amplicon lengths used in most studies might not be adequate to properly resolve the topology at the tips of the phylogeny. This is particularly important when examining patterns among sub-taxa, which at the genome level are defined at a cutoff of average nucleotide similarity of >95% [92, 93] while this cutoff is usually >97% of sequence similarity at the level of the 16 S rRNA gene [22]. While this should have no effect on the identification of HoS clades because their phylogenetic turnover should still be lower than that of outgroups, it might affect the identification of specific ASVs of interest within these clades. Thus full-length 16 S rRNA gene amplicons [94–96] or metagenome-assembled genomes in shotgun metagenomic studies might be used to construct phylogenetic trees with highly supported topologies near the tips to be used in our analytical framework.

## Supplementary information

Supplementary Information
